# Prognostic significance of HOXD4 protein expression in human ovarian cancers

**DOI:** 10.22038/IJBMS.2021.58396.12969

**Published:** 2021-11

**Authors:** Bo Yu, Xiaoqing Guo

**Affiliations:** 1 Department of General Surgery, The Second People’s Hospital of Lanzhou, Lanzhou 730000, China; 2 Department of Gynecology and Obstetrics, Affiliated Hospital of Gansu University of Traditional Chinese Medicine, Lanzhou, 730000, China

**Keywords:** Biomarker, Homeobox D4, Ovarian Neoplasms, Prognosis, Proliferation

## Abstract

**Objective(s):**

Ovarian cancer is the most common gynecological malignancy, ranking as the fifth leading cause of cancer-related deaths among females in the United States. Homeobox D4 (HOXD4) is a transcription factor belonging to the homeobox protein family, which plays a critical role in morphogenesis during embryo development. Here we aimed to study the HOXD4 expression in ovarian serous carcinoma (OSC) and determine its clinical significance.

**Materials and Methods:**

Real-time quantitative PCR and immunohistochemistry targeting human OSC tissues and adjacent ovarian tissues were performed to correlate the patterns of HOXD4 expression with clinical characteristics and survival outcomes. Cell lines and nude mice were used for verifying the role of HOXD4 in OSC.

**Results:**

HOXD4 protein was predominantly expressed in OSC tissues compared with nontumorous tissues. The correlation test demonstrated a significant correlation between HOXD4 with tumor FIGO stage. Univariate and multivariate analyses found that HOXD4 expression was associated with poorer overall survival. Furthermore, high expression of HOXD4 protein was observed in OSC cell lines in vitro. Finally, the oncogenic effect of HOXD4 was confirmed by cellular and xenograft experiments.

**Conclusion:**

HOXD4 protein expression may be associated with a poorer prognosis in OSC. The unfavorable prognostic value of HOXD4 in malignancies and its underlying mechanism are worthy of further investigation.

## Introduction

Ovarian cancer (OC) is acknowledged as the fifth leading cause of cancer-related deaths among females in the United States (1). The incidence of OC has increased during the past years worldwide (2). In 2015, there were 521,000 new cases diagnosed with OC in China, leading to approximately 225,000 related deaths (3). Although progressions on surgery, chemotherapy, radiotherapy, and immunotherapy have improved patient prognosis (4), the 5-year overall survival rate is less than 40% for those diagnosed with advanced stages (5). Epithelial OC accounts for 85% to 90% of malignant OCs, and serous ovarian cancer (OSC) is the predominant pathological type (6). OSC represents the most malignant type of OC, and the prognosis of OSC is far from satisfactory. Of note, there is no effective molecular target for OSC treatment; currently, surgery combined with postoperative chemotherapy is the first-line therapeutic procedure. The heterogenicity and unclear pathophysiologic mechanisms may partially explain the unsatisfied clinical outcomes. Therefore, it is urgently needed to develop effective biomarkers to improve detection, prognostic prediction, and treatment of OSC, which will help alleviate the disease burden.

***Homeobox D4 (HOXD4) belongs to the homeobox***

family proteins, which plays an important role in morphogenesis as a transcription factor. HOXD4 was originally identified for its regulatory function of providing cells with specific positional identities on the limbs (7).  The developmental-related role of HOXD4 can be partially reflected by the fact that its transgenic expression in neuronal progenitor cells resulted in death shortly after mice birth (8). Interestingly, dysregulated HOXD4 was also observed in pluripotent embryonal carcinoma (9), further highlighting its role in embryo development. Later HOXD4 was reported to be a regulator of proliferation and differentiation of hematopoietic cells. Van Scherpenzeel Thim *et al*. identified a germline Glu81-to-Val (E81V) mutation in the HOXD4 gene from two children with acute lymphoblastic leukemia; they hypothesized this missense mutation results in a partial loss of function, subsequently leading to childhood acute lymphoblastic leukemia (10). Besides, HOXD4 participates in adipogenesis, which can be down-regulated by PPARγ (11). Loss-of-function mutants and ectopic expression experiments revealed that HOXD4 is essential for cell segregation(12). Moreover, induction of HOXD4 is sufficient to induce both growth arrest and neuronal differentiation in neuroblastoma cells, which may be associated with downstream regulation of genes on cell cycle and differentiation (13). Nevertheless, our knowledge regarding the expression profile and function of HOXD4 in malignancies is limited. Till now, there is no study reporting the role of HOXD4 in OC. 

To investigate the correlation between HOXD4 and OSC progression, we initially compared mRNA and protein levels of HOXD4 in clinical OSC tissues and paired nontumorous ovarian tissues. The clinical significance of HOXD4 on predicting OSC overall survival was evaluated by univariate and multivariate analyses. Furthermore, we confirmed the tumor-related role of HOXD4 in OSC by *in vitro* and *in vivo* experiments. 

## Materials and Methods

***Ethics statement***

This study was approved by the Ethics Committee of the Second People’s Hospital of Lanzhou and written informed consent was obtained from each participant. All specimens were handled and made anonymous according to ethical and legal standards.

***Patients and specimens***

OSC tissues and nontumorous ovarian specimens were obtained from patients who had undergone surgery at the Second People’s Hospital of Lanzhou. All patients included in our clinical assays were diagnosed with OSC based on histopathological evaluation. This study contains two cohorts. The first cohort contains 17 paired samples that were flash-frozen in liquid nitrogen immediately after resection and stored at 

-80 °C for RT-qPCR tests. The second cohort contains 112 paired samples that were formalin-fixed and paraffin-embedded, which were used for immunohistochemistry assay and survival analyses. Patients with any of the following conditions were excluded: neoadjuvant therapy, with other serious diseases, or recurrent cancer. Clinical information of all 112 patients was available. The overall survival was defined as the time from surgery to death or the date of the last follow-up. The clinicopathological features of all patients are listed in Table 1. 

***Immunohistochemistry (IHC)***

IHC was conducted to evaluate HOXD4 expression in clinical tissue samples. Briefly, the tissue sections were deparaffinized, rehydrated, and then incubated with 3% hydrogen peroxide (H_2_O_2_) in methanol. Antigen retrieval was conducted by using ethylene-diamine-tetra acetic acid (EDTA) buffer. The tissue sections were blocked with 5% bovine serum (BSA) and then probed with anti-HOXD4 (#PA5-64441, Invitrogen) at 4 °C overnight. Secondary antibody was then added and incubated. The immunoreactivity was finally detected by using the diaminobenzidine (DAB) staining reagents as described by others (14). 

The IHC results were independently scored by two pathologists based on staining intensity and the percentage of positively stained cells. Staining intensity score was given as negative staining: 0**;** weak staining**:** 1**;** moderate staining: 2; and strong staining: 3. Percentage of positive cells was scored as 0–25%: 1; 26–50%: 2; 51–75%: 3; and >75%: 4. The immunoreactivity score was obtained by multiplying the two scores above, ranging from 0–12. 

***Total RNA extraction and quantitative PCR analysis (RT-qPCR)***

The mRNA isolation and RT-qPCR assays were conducted as previously described (15). Briefly, total RNA was extracted using Trizol reagent (Invitrogen, USA) according to the instructions. Then extracted RNA was synthesized to cDNA by the PrimeScript™ RT reagent Kit (Takara, Japan). Quantitative polymerase chain reaction (qPCR) was done using the SYBR Premix Ex Taq kit (Takara) on the Applied Biosystems. Data were calculated by the 2-ΔΔCt method using the endogenous control of GAPDH (16). The primers were as follows:

HOXD4-Forward: 5′-TTCTGGCCCTCAGTGAATGG-3′

HOXD4-Reverse: 5′-CTCGACACCCGCTAACAAATG-3′

GAPDH-Forward: 5′-TGCACCACCAACTGCTTAGC-3′

GAPDH-Reverse: 5′-GGCATGGACTGTGGTCATGAG-3′

***Cell culture and infection***

Ovarian surface epithelium Hs832 cell line and human OS cell lines (A2780, OVCAR-3, SKOV-3) were purchased from the Chinese Academy of Sciences (Shanghai, China). All cell lines were cultured at 37 °C with 5% CO_2_ in standard medium supplemented with 10% fetal bovine serum (FBS) and antibiotics. Lentiviruses to provide stable interference of HOXD4 (HOXD4-shRNA) and control-shRNA were synthesized by Amsbio (Cambridge, MA, USA). Briefly, A2780 cells were infected with interfering lentivirus according to the standard procedures (16). The infected cells were identified and selected using puromycin to obtain stable-infected cells. 

***Western blot***

The protein expression in cultured cells was tested by Western blot as previously described (18, 19). Briefly, cell lysates from cultured cells were quantified by a BCA kit and separated using 12% SDS-PAGE. Proteins were then transferred from the gel onto the PVDF membrane (Millipore, USA) followed by blocking with 5% BSA for 1 hr at room temperature. PVDF membranes were incubated with corresponding primary antibodies at 4 °C overnight. After incubation with peroxidase-conjugated secondary antibodies, the immunoreactivities were detected by adding ECL chemiluminescent solution and exposed on X-ray films. β-actin was used as an internal control.

***Cell proliferation analysis***

Cell proliferation was analyzed using MTT assay as we previously described (20). Briefly, stable-infected cells were seeded into a 96-well plate and cultured in standard conditions. The cells were incubated for 1, 2, 3, and 4 days. 20 μl of MTT (5 mg/ml) solution was added to each well and incubated for 4 hr. Then the supernatants were removed and 150 μl of DMSO reagent was added into each well. The absorbance value (OD) of each well was measured at 490 nm. Each experiment was conducted in triplicate.

***In vivo tumor model***

The animal study was approved by the Ethics Committee of the Second People’s Hospital of Lanzhou. The 4-week-old BALB/c female nude mice were used for establishing the subcutaneous tumor mice model. Briefly, nude mice (n=8) were injected subcutaneously with 2 × 10^6^ stable infected A2780 cells and cultured. Tumor size was measured using calipers every 5 days for 20 days. Tumor volume was calculated by the formula: (π x length x width^2^) / 6. After 20 days, all mice were sacrificed, and the xenografts were isolated for weighting (21). 

***Statistical analysis***

Statistical analyses were conducted using the SPSS 18.0 statistical software (SPSS Inc., Chicago, IL, USA). The associations between HOXD4 expression and clinicopathological parameters were analyzed using the Chi-square test. Overall survival curves were generated using the Kaplan-Meier method and log-rank test for comparison. Independent prognostic factors were estimated by the Cox proportional hazards stepwise regression model. All *P*-values were 2-sided and *P*<0.05 was considered statistically significant.

## Results

***Patient characteristics***

For the retrospective cohort containing 112 OSC cases, the median age was 54 and ranging from 41 to 76 years old. There were 39 cases (34.8%) with pathological differentiation grade I, 35 cases (31.3%) with grade II, and the other 38 cases (33.9%) with grade III. As for the lymph node status, 65 cases (58.0%) were designated as positive lymph node metastasis, while the other 47 cases (42.0%) designated negative lymph nodes. Accordingly, 35 cases (31.3%) were classified as FIGO stage I, 42 cases (37.5%) with stage II, 23 cases (20.5%) with stage III, and the other 12 cases (10.7%) with stage IV. The median follow-up time was 55 months, ranging from 2–114 months. By the end of the follow-up, 25 cases were dead. The 5-year overall survival of this cohort was 87.9%. 

***HOXD4 expression in OSC***

**Figure 1:**
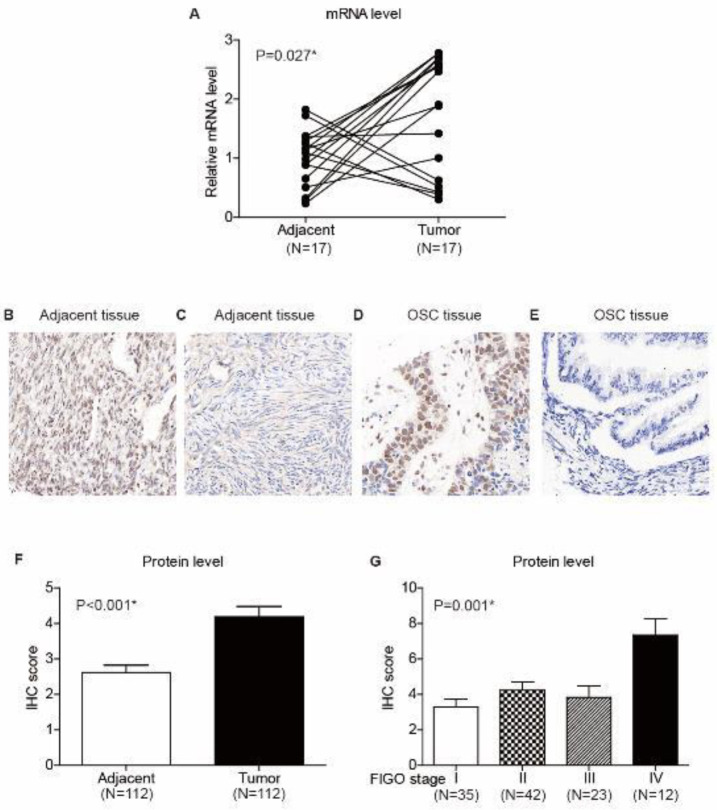
Expression of Homeobox D4 (HOXD4) in ovarian serous carcinoma (OSC) and adjacent tissues

The mRNA level of HOXD4 was firstly tested in 17 paired OSC and adjacent tissues (Figure 1A). As a result, *HOXD4* showed significantly higher levels in OSC samples than in normal adjacent specimens (*P*=0.027). We next evaluated the protein expression pattern of HOXD4 in FFPE tissues by IHC strategy (Figures 1B-1E). Accordingly, HOXD4 showed significant nucleus localization and exhibited distinct immunoreactivities in different tissue samples. Consistent with mRNA level, OSC tissues showed significantly higher HOXD4 protein levels than adjacent samples (*P*<0.001, Figure 1F). Moreover, cases with advanced FIGO stages showed higher HOXD4 protein levels (*P*=0.001, Figure 1G). 

**Figure 2:**
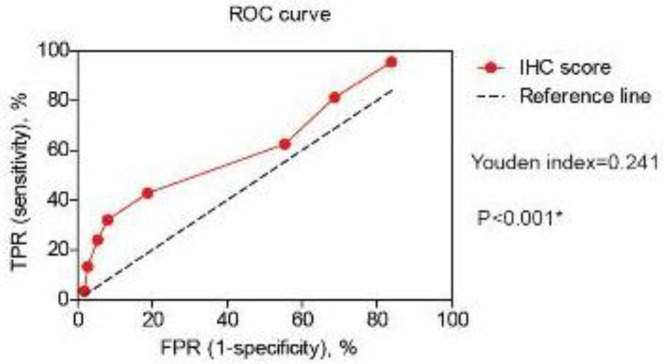
Receiver operating characteristic (ROC) curve of Homeobox D4 (HOXD4) expression in ovarian serous carcinoma (OSC) cohort

The immunoreactivity scores of HOXD4 were then subjected to a receiver operating characteristic (ROC) curve to obtain a cut-off value (Figure 2). Accordingly, the sample was grouped into high-HOXD4 expression (a score of >4, n=48) or low-HOXD4 expression (a score of ≤4, n=64). We further investigated the associations between protein expression of HOXD4 and clinicopathologic characteristics of OSC patients (Table 1). Based on the Chi-square test, higher HOXD4 levels were observed in patients with positive lymph nodes compared with those with negative lymph nodes (*P*=0.002). Similarly, higher HOXD4 expression was more frequently observed in patients with advanced FIGO stages (*P*=0.036). However, no statistically significant correlation was found between the expression of HOXD4 and other clinical factors, such as age (*P*=0.630), serum CA-125 (*P*=0.235), or pathological grade (*P*=0.068). The clinical associations suggested that HOXD4 may participate in OSC progression. 

***Prognostic significance of HOXD4 in OSC***

**Figure 3:**
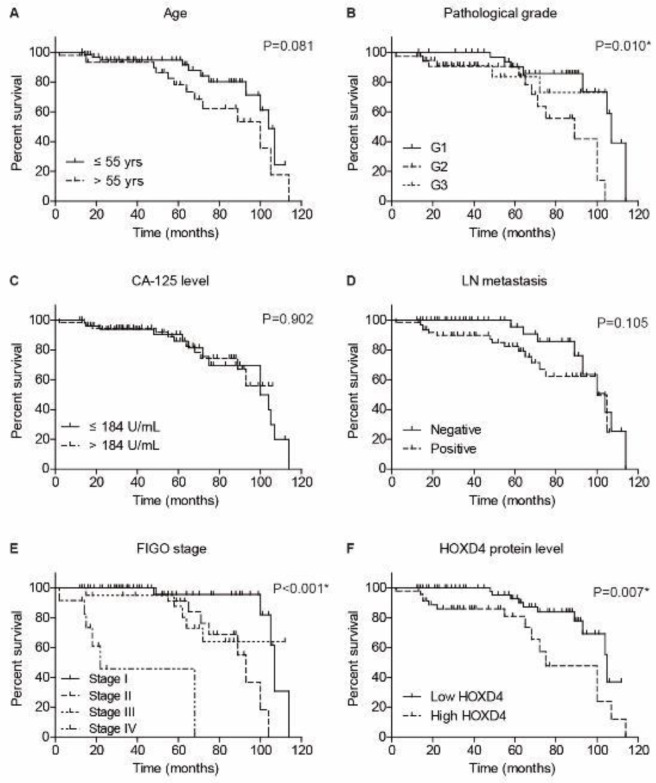
Overall survival analyses of enrolled ovarian serous carcinoma (OSC) patients

The prognostic significance of conventional clinicopathological characteristics and HOXD4 expression was tested by the Kaplan-Meier method (Figure 3). According to the univariate analyses, high HOXD4 expression was significantly associated with a poorer prognosis (*P*=0.007). The mean survival time of low-HOXD4 cases was 98.2 **± **3.5 months, while it was only 78.5 ± 6.6 months for those with high-HOXD4 levels (Table 2). Besides, patients with advanced pathological grade or advanced FIGO stage exhibited unfavorable prognosis (*P*=0.010 and *P*<0.001, respectively). We further subjected the significant prognostic factors into a multivariate Cox regression model (Table 3). As a result, HOXD4 was confirmed as an independent risk factor for OSC prognosis (*P*=0.004, HR=3.554, 95% CI: 1.493-8.459). As expected, the pathological grade (*P*=0.015, HR=3.796, 95% CI: 1.289-11.178) and FIGO stage (*P*=0.001, HR=2.252, 95% CI: 1.407-3.605) were also identified as independent prognostic factors.

***HOXD4 promotes OSC proliferation both in vitro and in vivo***

**Figure 4:**
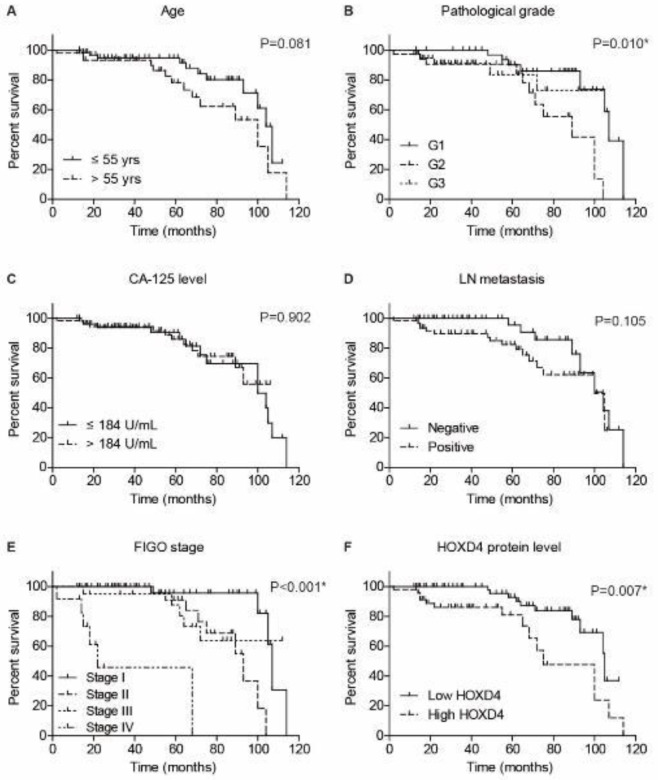
*In vitro* and* in vivo* evaluation of Homeobox D4 (HOXD4) function in ovarian serous carcinoma (OSC) progression

Since clinical data demonstrated the significant role of HOXD4 in OC patients. We next aimed to verify its tumor-related role in OSC cells. By testing its protein level in nontumorous Hs832 cells and three OSC cell lines (A2780. OVCAR-3, SKOV-3), we found that HOXD4 was up-regulated in all three OSC cell lines compared with that in Hs832 cells (Figure 4A). A2780 cells showed the highest endogenous HOXD4 level, thus were selected for knockdown experiments using specific shRNA by lentivirus infection (Figure 4B). After testing the knockdown efficiency, stable-infected cells were subjected to proliferation assay by MTT assays. As shown in Figure 4C, cells infected with HOXD4-shRNA exhibited an impaired proliferation capacity compared with those infected with control shRNA. 

Furthermore, we established the xenograft mice model by subcutaneously injecting stable infected A2780 cells. The xenograft growth curves showed that silencing HOXD4 can remarkably suppress tumor growth *in vivo* (Figure 4D). Consistently, the excised xenografts showed macroscopic difference and weight difference between the two groups (Figures 4E and 4F).

## Discussionon

Dysregulated expression of the HOXD4 transcription factor has been reported in several malignancies. For example, HOXD4 was overexpressed and positively correlated with oncogene-like character in spontaneously derived neoplastic canine mammary carcinoma cell models (19). Their data also revealed similar results in human breast cancer samples, suggesting its contribution to oncogenesis. Besides, HOXD4 was up-regulated in glioma tissues compared with normal brain tissues. Glioma patients with higher HOXD4 expression showed a significantly shorter survival than those with lower HOXD4 expression, indicating that HOXD4 may be a potential prognostic factor of gliomas (20). However, the expression pattern and clinical significance of HOXD4 in OC remain unclear. Here we first discovered that HOXD4 showed higher levels in OSC tissues than normal ovarian tissues on both mRNA and protein aspects. Moreover, higher HOXD4 was correlated with advanced tumor stages and served as an independent unfavorable prognostic factor. 

As a transcription factor, HOXD4 may exert oncogenic functions via multiple downstream genes. For example, HOXD4 may promote proliferation by up-regulating c-Myc and cyclin D1 in gastric cancer cells (21). Besides its downstream signaling pathways, the upstream modulating mechanism should also be considered. As reported in breast and gastric cancers, HOXD4 can be negatively regulated by microRNA-10b (22). Similarly, microRNA-10b plays tumor-suppressing roles in multiple cancers(23, 24). By using the shRNA infection strategy, our data demonstrated that silencing HOXD4 significantly attenuated the proliferation capacity of OSC cells, which is consistent with its reported role in other malignancies. Of note, our study initially provided *in vivo* evidence on the tumor-related role of HOXD4 in OC progression. Knockdown of HOXD4 resulted in a remarkable impaired growth process of xenografts in nude mice. 

Our study has several limitations. Firstly, the mRNA level of HOXD4 was analyzed in small sample size and we did not evaluate its correlation with patients’ characteristics. Here we focused more on its protein level and clinical significance. Secondly, all patients were from a single medical center, thus may contain regional bias. Thirdly, our study aimed to illustrate the prognostic value and oncogenic effects of HOXD4 on OSC phenotype, therefore we did not touch deeply on its functional mechanisms. Further systematic high throughput experiments will be necessary to illuminate the signaling mechanisms of HOXD4 in malignancies.

**Table 1:** Characteristics of the ovarian serous carcinoma (OSC) patients and associations with Homeobox D4 (HOXD4) expression level

**Variable**	**Cases**	**HOXD4 expression**		***P*** **-value**
	**(n = 112)**	**Low (n = 64)**	**High (n = 48)**	
**Age (years)**		54.9 ± 8.5	55.7 ± 8.9	0.630
**CA-125 (U/ml)**		187.7 ± 34.1	179.8 ± 34.8	0.235
**Pathological grade**				0.068
G1	39	28	11	
G2	35	18	17	
G3	38	18	20	
**LN metastasis**				0.002*
Negative	47	35	12	
Positive	65	29	36	
**FIGO stage**				0.036*
I	35	22	13	
II	42	22	20	
III	23	17	6	
III	12	3	9	

Note: *P<0.05 by the Chi-square test

(A) RT-qPCR assays showed the mRNA levels of HOXD4 in OSC and adjacent nontumorous tissues

(B) Representative high HOXD4 immunoreactivity in adjacent ovarian tissues (IHC score=12)

(C) Representative low HOXD4 immunoreactivity in adjacent ovarian tissues (IHC score=1)

(D) Representative high HOXD4 immunoreactivity in OSC tumor tissues (IHC score=12)

(E) Representative low HOXD4 immunoreactivity in OSC tumor tissues (IHC score=0)

(F) Immunohistochemistry (IHC) scores of HOXD4 in OSC tissues and paired adjacent tissues were summarized, which exhibited a higher average score in OSC tumor tissues

(G) IHC scores of HOXD4 in patients with different FIGO stages were compared by one-way ANOVA test, indicating a positive correlation between HOXD4 and tumor progression

ROC curve was generated to define a cutoff value for distinguishing low-HOXD4 and high-HOXD4 patients. Accordingly, the cutoff IHC score was 4 (*P*<0.001)

Predictive roles of age (A), pathological grade (B), preoperative serum CA-125 level (C), lymph node metastasis (D), FIGO stage (E), and Homeobox D4 (HOXD4) level (F) were tested by the Kaplan-Meier method. * indicates *P*<0.05 by log-rank test

**Table 2:** Univariate analysis for the overall survival of ovarian serous carcinoma (OSC) patients

**Variable**	**Cases**	**Overall survival**		***P*** **-value**
	**(n = 112)**	**Mean ± SD ** **(Months)**	**5-year (%)**	
**Age (years)**				0.081
≤ 55 yrs	66	95.6 ± 3.9	91.5%	
> 55 yrs	46	84.2 ± 5.8	78.4%	
**Pathological grade**				0.010*
G1	39	101.1 ± 4.2	93.3%	
G2	35	79.1 ± 6.1	86.2%	
G3	38	88.8 ± 6.2	83.5%	
**CA-125 (U/ml)**				0.902
≤ 184 U/ml	54	91.4 ± 4.9	93.8%	
> 184 U/ml	58	89.0 ± 4.3	83.4%	
**LN metastasis**				0.105
Negative	47	97.9 ± 4.3	95.5%	
Positive	65	84.3 ± 4.6	82.4%	
**FIGO stage**				<0.001*
I	35	105.2 ± 3.7	95.7%	
II	42	87.1 ± 4.5	91.1%	
III	23	92.4 ± 7.3	87.7%	
III	12	39.6 ± 9.9	45.8%	
**HOXD4 expression**				0.007*
Low	64	98.2 ± 3.5	92.8%	
High	48	78.5 ± 6.6	81.0%	

Note: *P<0.05 by log-rank test

**Table 3:** Multivariate analysis for the overall survival of ovarian serous carcinoma (OSC) patients

**Variable**	**Hazard ratio**	**95% CI**	***P*** **-value**
**Age** (>55 yrs vs ≤ 55 yrs)	1.365	0.585-3.185	0.472
**Pathological grade** (G2-G3 vs G1)	3.796	1.289-11.178	0.015*
**FIGO stage** (III-IV vs I-II)	2.252	1.407-3.605	0.001*
**HOXD4 expression** (High vs low)	3.554	1.493-8.459	0.004*

Note: *P<0.05 by Cox regression test

(A) Immunoblotting test demonstrated increased HOXD4 in human OSC cell lines (A2780. OVCAR-3, SKOV-3) compared with noncancerous ovarian epithelial cell line (Hs832)

(B) Knockdown assay was conducted by infecting lentivirus shRNAs targeting HOXD4 in A2780 cells, using scramble shRNA as control. The knockdown efficiency was tested by Western blotting

(C) Silencing HOXD4 resulted in a significant inhibition on the proliferation process of A2780 cells, as revealed by MTT assays

(D) By plotting the tumor growth curve, we found that HOXD4-knockdown significantly attenuated OSC growth in subcutaneous xenograft models

(E) Macroscopic image of isolated xenografts from HOXD4-shRNA and control shRNA groups

(F) Isolated xenografts were weigted, which indicated a significant decrease in the HOXD4-shRNA group compared with the control group

*P<0.05 by Student’s t-test

## Conclusion

Taken together, our findings demonstrated that HOXD4 might serve as a promising biomarker for prognosis prediction of OSC.

## Data Availability

 Data is available upon request.

## Authors’ Contributions

BY conducted the statistical analysis and wrote this manuscript; XG designed this project and conducted experimental assays.

## Funding

None.

## Conflicts of Interest

None.
